# Review on “Long-Dan”, one of the traditional Chinese medicinal herbs recorded in Chinese pharmacopoeia

**DOI:** 10.1007/s13659-011-0043-3

**Published:** 2012-02-13

**Authors:** Yan-Ming Wang, Min Xu, Dong Wang, Hong-Tao Zhu, Chong-Ren Yang, Ying-Jun Zhang

**Affiliations:** 1 State Key Laboratory of Phytochemistry and Plant Resources in West China, Kunming Institute of Botany, Chinese Academy of Sciences, Kunming, 650201 China; 2 Graduate University of Chinese Academy of Sciences, Beijing, 100049 China; 3 Weihe Biotech Laboratory, Yuxi, 653100 China

**Keywords:** Long-Dan, Chinese Pharmacopoeia, herbal textual study, chemistry, bioactivity, quality control

## Abstract

“Long-Dan” is an important traditional Chinese medicinal (TCM) herb used widely for the treatment of inflammation, hepatitis, rheumatism, cholecystitis, and tuberculosis. In the Chinese Pharmacopoeia, the roots and rhizomes of four species from the genus *Gentiana* (Gentianaceae) are recorded as the original materials of “Long-Dan”, called Gentianae Radix et Rhizoma. The species included *G. manshurica, G. scabra, G. triflora* and *G. rigescens*, which are distributed in different areas of China. Though iridoid and secoiridoid glucosides were reported as the main constituents in “Long-Dan”, these four different species also resulted in different minor components, which may related to their pharmacological activities. Herein, we summarized the herbal textual study, distribution, chemical constituents, biological investigation and quality control of the recorded “Long-Dan” origins in Chinese Pharmacopoeia during the period 1960 to 2011.

## Introduction

"Long-Dan" is a well-known traditional Chinese medicinal (TCM) herb commonly used for curing inflammation, hepatitis, rheumatism, cholecystitis, and tuberculosis. In the Chinese Pharmacopoeia, the roots and rhizomes of four species from the genus *Gentiana* (Gentianaceae), including *G. manshurica* Kitag. (Tiao-Ye-Long-Dan), *G. scabra* Bge. (Cu-Cao-Long-Dan, Long-Dan), *G. triflora* Pall. (San-Hua-Long-Dan) and *G. rigescens* Franch. (Dian-Long-Dan), are recorded as the raw materials of "Long-Dan" and called Gentianae Radix et Rhizoma.[1] These four *Gentiana* species are distributed in different areas of China. Though iridoid and secoiridoid glucosides, e.g. loganic acid, gentiopicroside, sweroside and swertiamarin were reported as the main constituents, different species also resulted in their different minor components. Modern pharmacological studies indicated that "Long-Dan" had various bioactivities, such as hepatoprotective, anti-inflammatory, analgesic, anti-proliferative, and anti-microbial effects. Owing to these bioactivities and chemical constituents, much attention has been paid to different "Long-Dan" species. A review described the phytochemicals and biological activities of *Gentiana* species from 1960 to June of 2009,[2] however, no one focused on the recorded "Long-Dan" origins in Chinese Pharmacopoeia from various aspects. The present article summarized the herbal textual study, distribution, phytochemical progress, biological activities, as well as the quality control of the four recorded "Long-Dan" origins during the period 1960 to 2011. It is hopeful that our crude remark may bring out some good idea with the development of new strategies for traditional medicine in the future.

## 1 Herbal Textual Study

"Long-Dan" has been used as a medicinal herb since ancient times. It was firstly recorded on "Shennong Bencao Jing" called "Ling-You" from more than 2000 years ago. Several variant names, e.g. "Cao-Long-Dan", "Long-Dan-Cao", "Ku-Cao" and "Dan-Cao" had been used and regarded as the synonyms of "Long-Dan" in ancient times, according to the historical records. All of them were relevant to the bitterness of the herb.[3] In "Mingyi Bielu", it recorded that "Long-Dan" grew in Shandong province of China, whose roots were picked in February, August, November, and December, and dried in shade, while "Jiuhuang Bencao" described that "Long-Dan" was found in Henan province. "Zhiwu Mingshi Tukao" (Illustrated Book on Plants in Qing Dynasty) recorded that "Dian-Long-Dan" (*G. rigescens*) was growing in the mountains of Yunnan province.[3] Since "Tiao-Ye-Long-Dan"

*To whom correspondence should be addressed. E-mail: xumin@mail.kib.ac.cn; zhangyj@mail.kib.ac.cn

(*G. manshurica*) was the largest and most widely distributed species of "Long-Dan", it should be the mostly used variety in ancient times.[4]

The earliest record about the function of "Long-Dan" was in "Shennong Bencao Jing", in which it was characterized by bitter in taste and cold in nature. "Mingyi Bielu" described "Long-Dan" as innoxious and great cold in nature. About the channel tropism, "Long-Dan" was ascribed to the meridian of liver, kidney, and urinary bladder in "Leigong Paozhi Yaoxing Jie" and remarked by "Yaoxing Jie". In addition to the bitterness, "Long-Dan" also possessed the taste of sour, which contributed to the digestion of stomach, as those accounted in "Yixue Zhongzhong Canxi Lu". "Yaoxing Fu" wrote that it could remove the heat of liver and the damp-abscess of "Xiajiao" concerning of the organs of liver and kidney. In "Bencao Gangmu" (Outline Treatise of Medical Herbs, compiled by Shi-Zhen Li), "Long-Dan" was recorded to be used for the treatment of sore throat and night sweats, while in "Depei Bencao", it was ascribed to cure jaundice, relieve diarrhea, and heal bulbar conjunctiva congestion. Chen Shiduo noted that "Long-Dan-Cao" was used especially for eliminating dampness through diuresis and curing jaundice in "Ben-cao Xinbian".[5]

It is noted that the records about the function of "Long-Dan" in ancient medical books were in accordance with the modern clinical application of this herb for curing inflammation, hepatitis, rheumatism, cholecystitis, and tuberculosis.

## 2 Distribution

Among the four original plants (*G. manshurica*, *G. scabra*, *G. triflora*, and *G. rigescens*) of Gentianae Radix et Rhizoma in Chinese Pharmacopoeia,, the former three species, are mainly distributed in the northeast of China, used to be called as "Guan-Long-Dan", while the last one called as "Dian-Long-Dan" is mainly growing in the southwest of China, particularly in the mountainous areas of Yunnan province.[1]

*G. manshurica* grows in between the edge to the clearings of forest, hillsides, roadsides, meadows, and grassland near to the cropland with altitude at 200–1700 m. It is mainly distributed in northeast of China, and south part of Shandong, Shanxi, and Henan province. Some populations are also growing in the central part of Shanxi and Hubei provinces, and the west regions of Hunan, Guangxi, Guangdong, Jiangxi, and Zhejiang provinces.[4]

*G. scabra* could be found mainly in the meadows, forest edge, and scrub or river meadow. It is distributed primarily in the three Northeast China provinces, the northeastern area of Inner Mongolia, and northern regions of Fujian and Zhejiang provinces.[4]

*G. triflora*, found in meadows, glade, or shrubs with altitude ca. 200–1500 m is mainly distributed in Heilongjiang, Jilin, and Inner Mongolia of China.[4]

*G. rigescens* grows in the slopes grass or short grass with altitude 800–3000 m. This species is native to Yunnan, Sichuan, Guizhou, and the western part of Guangxi province.[4]

All of these species were found in the wild with the specimen's picker and pressed for subsequent identification in herbaria. However, with the long-term and large-scale dredging, wild production of "Long-Dan" was severely reduced. Therefore, some *Gentiana* species have been introduced into cultivation, among which *G. rigescens* and *G. scabra* are two commonly cultivated species in recent years.[6,7] *G. rigescens* and *G. scabra* are the main variety in the Southwestern and Northeastern herbal markets, respectively.

## 3 Chemical Constitutions

A series of iridoids were isolated and characterized as the main constituents in "Long-Dan", together with triterpenes, lignans, flavonoids, coumarins, and other types of compounds, summarized totally as 96 compounds. Their structures were shown below, and their names and the corresponding plant sources were organized in Table1.

**3.1 Iridoids:** As the major chemical constituents, 30 iridoids (compounds **1**–**30**) were reported from "Long-Dan". Among them, three secoiridoidal glucosides, gentiopicroside (**1**), swertiamarin (**2**), and sweroside (**3**), and one iridoidal glucoside, loganic acid (**23**) are not only the four main components and active ingredients in "Long-Dan", but also widely distributed in the whole family of Gentianaceae.[8] In addition, some new iridoids were reported from *G. rigescens* and *G. scabra*. For example, six new secoiridoid glycosides, gentiascabraside A (**8**) with a methoxyl group occurred at C-3,[9] 4'''-*O*-*β*-D-glucopyranosyltrifloroside (**15**),[10] 4'''-*O*-*β*-D-glucopyranosylscabraside (**17**),[10] and compounds **20**–**22**[9] characterized as gentiopicroside with 3–5 additional *β*-D-glucopyranosyl moieties attached to the C-6′ of inner glucopyranosyl unit, were isolated from the roots and rhizomes of *G. scabra*. Gentiorigenoside A (**29**), whose C-11 was esterified with an ethyl group, and C-7 and C-6′ were acetylated, was a new secoiridoidal glucoside from the roots and rhizomes of *G. rigescens*.[11]

Compared with those from the other *Gentiana* species, the iridoids from the recorded "Long-Dan" origins in Chinese Pharmacopoeia were not very diverse, majority gentiopicroside derivatives (**1**, **4**–**6**, **8**–**11**, **14**, **20**–**22**) and sweroside derivatives (**3**, **7**, **13**, **15**–**19**). Only one kingiside derivative (**25**), three carbocyclic iridoids (**23**–**24**, **26**) and two secologanic acid derivatives (**29** and **30**) were reported from these recorded "Long-Dan" origins, in which four iridoids, gentiopicroside (**1**), swertiamarin (**2**), sweroside (**3**), and loganic acid (**23**) were the common chemical composition. The few gentiopicroside derivatives, **8**–**10**, oxygenated by OH or OMe at C-3, C-4 and C-6 positions, should be the typical chemical composition of *G. scabra*.

**3.2 Triterpenes:** Triterpenes also widely occurred in "Long-Dan".[12] To date, 27 triterpenes (**31**–**57**) were identified from *G. rigescens* and *G. scabra*.

The natural occurrence of chiratane triterpenoids was extremely rare. Chirat-17(22)-en-3-one (**49**), isolated from the rhizomes and roots of *G. scabra* in 2002, was the first example of a chiratane triterpenoid having a $\Delta^{17(22)}$ double bond.[13] Guo et al. also reported this kind of compounds from the roots of *G. scabra* in 2011.[14] Chirat-16-en-3-one (**48**) and 17*β*,21*β*-epoxyhopan-3-one (**55**) possessing a ketone group at C-3, have been synthesized in 1990, however, the first isolation of

**Table 1. Chemical constituents and their plant sources**

| No. | Compounds | Plant sources | Parts of plants | Ref. |
|---|---|---|---|---|
| | **Iridoids** | | | |
| **1** | Gentiopicroside | *G. manshurica* | Root | 44 |
| | | *G. scabra* | Root | 45,14 |
| | | *G. scabra* | Rhizomes | 10 |
| | | *G. triflora* | Root | 46,47 |
| | | *G. triflora* | Aerial part | 48 |
| | | *G. rigescens* | Root | 11,7 |
| | | *G. rigescens* | aerial part | 16 |
| **2** | Swertiamarin | *G. manshurica* | Root | 42 |
| | | *G. scabra* | Root | 43 |
| | | *G. triflora* | Root | 47,50 |
| | | *G. rigescens* | Root | 7,11 |
| **3** | Sweroside | *G. manshurica* | Root | 44 |
| | | *G. scabra* | Root | 45 |
| | | *G. triflora* | Root | 47 |
| | | *G. rigescens* | Root | 7,11 |
| **4** | 6'-*O*-*β*-D-Glucosylgentiopicroside | *G. scabra* | Root | 51 |
| | | *G. triflora* | Root | 47 |
| | | *G. rigescens* | Root | 11 |
| **5** | 4'-*O*-*β*-D-Glucosylgentiopicroside | *G. scabra* | Root | 51 |
| **6** | Olivieroside | *G. scabra* | Root | 51 |
| **7** | 8-Hydroxy-10-hydrosweroside | *G. scabra* | Root | 49 |
| **8** | Gentiascabraside A | *G. scabra* | Rhizome & root | 9 |
| **9** | 6*β*-Hydroxyswertiajaposide A | *G. scabra* | Rhizome & root | 9 |
| **10** | Swertiajaposide A | *G. scabra* | Rhizome & root | 9 |
| **11** | Amarogentin | *G. manshurica* | Root | 44 |
| **12** | Amaroswerin | *G. manshurica* | Root | 44 |
| **13** | 2'-(*o*,*m*-Dihydroxybenzyl)sweroside | *G. scabra* | Root | 45 |
| | | *G. triflora* | Root | 47 |
| | | *G. rigescens* | Root | 7,11 |
| **14** | Gentiotrifloroside | *G. triflora* | Root | 47 |
| **15** | 4'''-*O*-*β*-D-Glucosyltrifloroside | *G. scabra* | Rhizomes | 10 |
| **16** | Trifloroside | *G. scabra* | Rhizomes | 10 |
| | | *G. scabra* | Root | 49 |
| | | *G. triflora* | Root | 52 |
| **17** | 4'''-*O*-*β*-D-Glucosylscabraside | *G. scabra* | Rhizomes | 10 |
| **18** | Scabraside | *G. scabra* | Rhizomes | 10 |
| | | *G. scabra* | Root | 54 |
| **19** | Rindoside | *G. scabra* | Root | 53 |
| **20** | Scabrans $G_3$ | *G. scabra* | Rhizome & root | 9 |
| **21** | Scabrans $G_4$ | *G. scabra* | Rhizome & root | 9 |
| **22** | Scabrans $G_5$ | *G. scabra* | Rhizome & root | 9 |
| **23** | Lognic acid | *G. scabra* | Root | 53 |
| | | *G. triflora* | Root | 47 |
| | | *G. rigescens* | Root | 7,11 |
| | | *G. rigescens* | Aerial part | 16 |
| **24** | 6'-*O*-*β*-D-Glucosylloganic acid | *G. scabra* | Root | 55 |
| | | *G. rigescens* | Aerial part | 36 |
| **25** | 8-Epikingiside | *G. scabra* | Root | 55 |
| **26** | Gentianaside | *G. scabra* | Root | 55 |
| **27** | 1-*O*-*β*-D-Glucosyl-4-epiamplexine | *G. scabra* | Rhizome & root | 9 |
| **28** | 1-*O*-*β*-D-Glucosyl-amplexine | *G. scabra* | Root | 51 |
| **29** | Gentiorigenoside A | *G. scabra* | Root | 11 |
| **30** | Secologanoside | *G. scabra* | Root | 11 |
| | **Triterpenes** | | | |
| **31** | Gentirigenic acid | *G. rigescens* | Root | 36 |
| **32** | Gentirigeoside A | *G. rigescens* | Root | 36 |
| **33** | Gentirigeoside B | *G. rigescens* | Root | 36 |
| **34** | Gentirigeoside C | *G. rigescens* | Root | 36 |

| | | | | |
|---|---|---|---|---|
| **35** | Gentirigeoside D | *G. rigescens* | Root | 36 |
| **36** | Gentirigeoside E | *G. rigescens* | Root | 36 |
| **37** | *α*-Amyrin | *G. scabra* | Rhizome & root | 13 |
| | | *G. rigescens* | Root | 7 |
| | | *G. rigescens* | Aerial part | 16 |
| **38** | *α*-Amyrin palmitate | *G. scabra* | Root | 14 |
| | | *G. rigescens* | Root | 7 |
| | | *G. rigescens* | Aerial part | 48 |
| **39** | Ursolic acid | *G. scabra* | Aerial part | 56 |
| | | *G. rigescens* | Root | 7 |
| | | *G. rigescens* | Aerial part | 16 |
| **40** | *β*-Amyrin | *G. scabra* | Aerial part | 56 |
| | | *G. rigescens* | Root | 7 |
| | | *G. rigescens* | Aerial part | 16 |
| **41** | *β*-Amyrin palmitate | *G. rigescens* | Root | 7 |
| | | *G. rigescens* | Aerial part | 16 |
| **42** | *β*-Amyrin acetate | *G. scabra* | Aerial part | 56 |
| **43** | Oleanolic acid | *G. scabra* | Root | 15 |
| | | *G. scabra* | Aerial part | 56 |
| | | *G. rigescens* | Root | 7 |
| | | *G. rigescens* | Aerial part | 16 |
| **44** | Uvaol 3-*O*-linoleate | *G. scabra* | Root | 57 |
| **45** | Uvaol 3-*O*-stearate | *G. scabra* | Root | 57 |
| **46** | Erythrodiol 3-*O*-linoleate | *G. scabra* | Root | 57 |
| **47** | Erythrodiol 3-*O*-stearate | *G. scabra* | Root | 57 |
| **48** | Chirat-16-en-3-one | *G. scabra* | Rhizome & root | 13 |
| **49** | Chirat-17(22)-en-3-one | *G. scabra* | Rhizome & root | 13 |
| **50** | Chiratenol | *G. scabra* | Rhizome & root | 13 |
| **51** | (20*S*)-Dammara-13(17),24-dien-3-one | *G. scabra* | Rhizome & root | 13 |
| **52** | (20*R*)-Dammara-13(17),24-dien-3-one | *G. scabra* | Rhizome & root | 13 |
| **53** | Lupeol | *G. scabra* | Rhizome & root | 13 |
| | | *G. rigescens* | Root | 7 |
| **54** | Lupeol palmitate | *G. rigescens* | Root | 7 |
| **55** | 17*β*,21*β*-Epoxyhopan-3-one | *G. scabra* | Rhizome & root | 13 |
| **56** | Hop-17(21)-en-3-one | *G. scabra* | Rhizome & root | 13 |
| **57** | Hop-17(21)-en-3*β*-ol | *G. scabra* | Rhizome & root | 13 |
| | **Lignans** | | | |
| **58** | *L*-Sesamin | *G. scabra* | Root | 15 |
| **59** | (−)-Syringaresinol-*O*-*β*-D-glucoside | *G. rigescens* | Root | 20 |
| **60** | (−)-Pinoresinol-*O*-*β*-D-glucoside | *G. rigescens* | Root | 20 |
| **61** | Liriodendrin | *G. rigescens* | Root | 7,20 |
| **62** | (−)-7*R*,8*S*-Dehydro-diconiferyl alcohol-4,9'-di-*O*-*β*-D-glucoside | *G. rigescens* | Root | 20 |
| **63** | (−)-7*R*,8*S*-Dehydro-diconiferyl alcohol-4-*O*-*β*-D-glucoside | *G. rigescens* | Root | 20 |
| **64** | Lariciresinol-4'-*O*-*β*-D-glucoside | *G. rigescens* | Root | 20 |
| **65** | Tortoside B | *G. rigescens* | Root | 20 |
| | **Coumarins** | | | |
| **66** | Pranfefin | *G. scabra* | Root | 15 |
| | **Flavanoids** | | | |
| **67** | Saponarin | *G. rigescens* | Aerial part | 16 |
| **68** | Isosaponarin | *G. rigescens* | Aerial part | 16 |
| **69** | Isoorientin | *G. rigescens* | Aerial part | 16 |
| **70** | Isoorientin-3'-*O*-*β*-D-glucoside | *G. rigescens* | Root | 20 |
| **71** | Kaempferol | *G. scabra* | Root | 15 |
| | **Others** | | | |
| **72** | Gentiside A | *G. rigescens* | Root | 17 |
| **73** | Gentiside B | *G. rigescens* | Root | 17 |
| **74** | Gentiside C | *G. rigescens* | Root | 18 |
| **75** | Gentiside D | *G. rigescens* | Root | 18 |
| **76** | Gentiside E | *G. rigescens* | Root | 18 |
| **77** | Gentiside F | *G. rigescens* | Root | 18 |
| **78** | Gentiside G | *G. rigescens* | Root | 18 |

| | | | | |
|---|---|---|---|---|
| **79** | Gentiside H | *G. rigescens* | Root | 18 |
| **80** | Gentiside I | *G. rigescens* | Root | 18 |
| **81** | Gentiside J | *G. rigescens* | Root | 18 |
| **82** | Gentiside K | *G. rigescens* | Root | 18 |
| **83** | 6-Demethoxy-7-methylcapillarisn | *G. scabra* | Aerial part | 56 |
| **84** | 2,3-Dihydroxy benzoic acid methyl ester 3-*O*-*β*-D-glucosyl-(1→6)-*β*-D-glucoside | *G. rigescens* | Root | 20 |
| **85** | 2,5-Dihydroxyl benzo5-*O*-*β*-D-xylosyl-(1→6)-*O*-*β*-D-glucoside | *G. rigescens* | Root | 20 |
| **86** | 4-*O*-*β*-D-Glucosyl-glicosyringic acid | *G. rigescens* | Root | 7 |
| **87** | 2-Hydroxy-3-methoxybenzoic acid glucose ester | *G. scabra* | Root | 19 |
| | | *G. rigescens* | Root | 20 |
| **88** | 3,5-Dimethoxy-4-hydroxybenzyl alcohol 4-*O*-*β*-D-glucoside | *G. rigescens* | Root | 20 |
| **89** | Vanillyl alcohol-*O*-*β*-D-glucoside | *G. rigescens* | Root | 20 |
| **90** | 4-Hydroxy-3-methoxyphenyl-*O*-*β*-D- xylosyl-(1→6)-*O*-*β*-D-glucoside | *G. rigescens* | Root | 20 |
| **91** | 3-*O*-*β*-D-Glycosylcaffeate | *G. rigescens* | Root | 20 |
| **92** | Oct-1-en-3-yl arabinosyl-(1→6)-*O*-*β*-D- glucoside | *G. rigescens* | Root | 20 |
| **93** | *β*-Sitosterol | *G. scabra* | Aerial part | 56 |
| | | *G. rigescens* | Aerial part | 16 |
| | | *G. rigescens* | Root | 7 |
| **94** | Daucosterol | *G. scabra* | Root | 45,14 |
| | | *G. rigescens* | Root | 7 |
| | | *G. rigescens* | Aerial part | 16 |
| **95** | Cholesterol | *G. scabra* | Root | 14 |
| **96** | *β*-Gentiobiose | *G. rigescens* | Root | 7 |

these two compounds from natural source was from *G. scabra* in 2002.[13] From the roots of *G. rigescens*, five new dammarane-type triterpenoid glycosides, gentirigeosides A–E (**32**–**36**), together with new dammarane-type aglycone, gentirigenic acid (**31**), were obtained. It was the first time to report triterpenoids with 20*S*,25-epoxy dammarane skeleton from family Gentianaceae.[36]

Triterpenes in the four recorded "Long-Dan" origins were much diverse. To date, 32 triterpenoids were reported from the genus *Gentiana*,[2] including 27 ones from the recorded "Long-Dan" origins in Chinese Pharmacopoeia. Meanwhile, triterpenes from different "Long-Dan" origins showed major differences. 20*S*,25-Epoxy dammaranes were the typical triterpenoid components in *G. rigescens*.[36]

**3.3 Lignans:** Compounds **58**–**65** were lignans derived from these four species of "Long-Dan". These compounds were mainly yielded from *G. rigescens*,[7,20] expect that *L*-sesamin (**58**) was derived from the roots of *G. scabra*.[15]

**3.4 Coumarins:** Compound **66** isolated from the roots of *G. scabra* was the only coumarin in "Long-Dan". It was separated and reported from the genus *Gentiana* for the first time.[15]

**3.5 Flavonoids:** To June of 2009, 60 flavonoids were identified in the genus *Gentiana*.[2] However, only five ones (**67**–**71**) were derived from the recorded "Long-Dan" origins (*G. rigescens* and *G. scabra*). The former four were *C*-glycosylflavones[16,20], while compound **71** was a flavonol.[15] It may be due to the different plant parts of 'Long-Dan". The roots and rhizomes are recorded for medicinal uses of "Long-Dan". Flavonoids were chiefly found in the aerial part of *G. rigescens*.[16]

**3.6 Others:** Compounds **72**–**96** were other type of compounds from *G. rigescens* and *G. scabra*. These ingredients comprised alkyl 2,3-dihydroxy-benzoates, phenolic glycoside, sterols, and disaccharides.

Gao et al reported a novel class of neuritogenic compounds from *G. rigescens*, namely gentisides A–K (**72**–**82**). These compounds were potent inducers of neurite outgrowth on PC12 cells. Their structure-activity relationships revealed that the length of alkyl chain was important for the activity, but the structural diversity at the end of the alkyl chain was not.[17,18] As the minor chemical constituents, 2,3-di-hydroxy benzoic acid methyl ester 3-*O*-*β*-D-glucopyranosyl-(1→6)-*β*-D-glucopyranoside (**84**) and 2,5-dihydroxyl benzofuran 5-*O*-*β*-D-xylopyranosyl-(1→6)-*O*-*β*-D-glucopyranoside (**85**) were two new phenolic glycosides derived from the roots of *G. rigescens*, and compound **84** also showed antifungal activities.[20]

## 4 Pharmacological Studies

**4.1 Hepatoprotective Effect:** "Long-Dan" is a famous hepatic used TCM herb for the treatment of hepatitis. The water extracts of *G. scabra* showed remarkable liver protective action by protecting the membranes of hepatocytes, inhibiting the immunoreaction occurred in liver, promoting the function of phagocytes, or enhancing the treatment for foreign body in damaged liver.[21] According to the experiment of Wang, hepatoprotective effects of *G. manshurica* against acetaminophen-induced acute toxicity were mediated either by preventing the decline of hepatic antioxidant status or its direct anti-apoptosis capacity.[22] Ethanolic extracts of *G. manshurica* showed protective effects on acute liver injury in mice induced by D-GalN and LPS.[23]

It's reported that the basic hepatoprotective ingredient was gentiopicroside (**1**). Gentiopicroside could not only reduce liver lipid peroxidation (LPO) of fasted mice and against $CCl_4$-

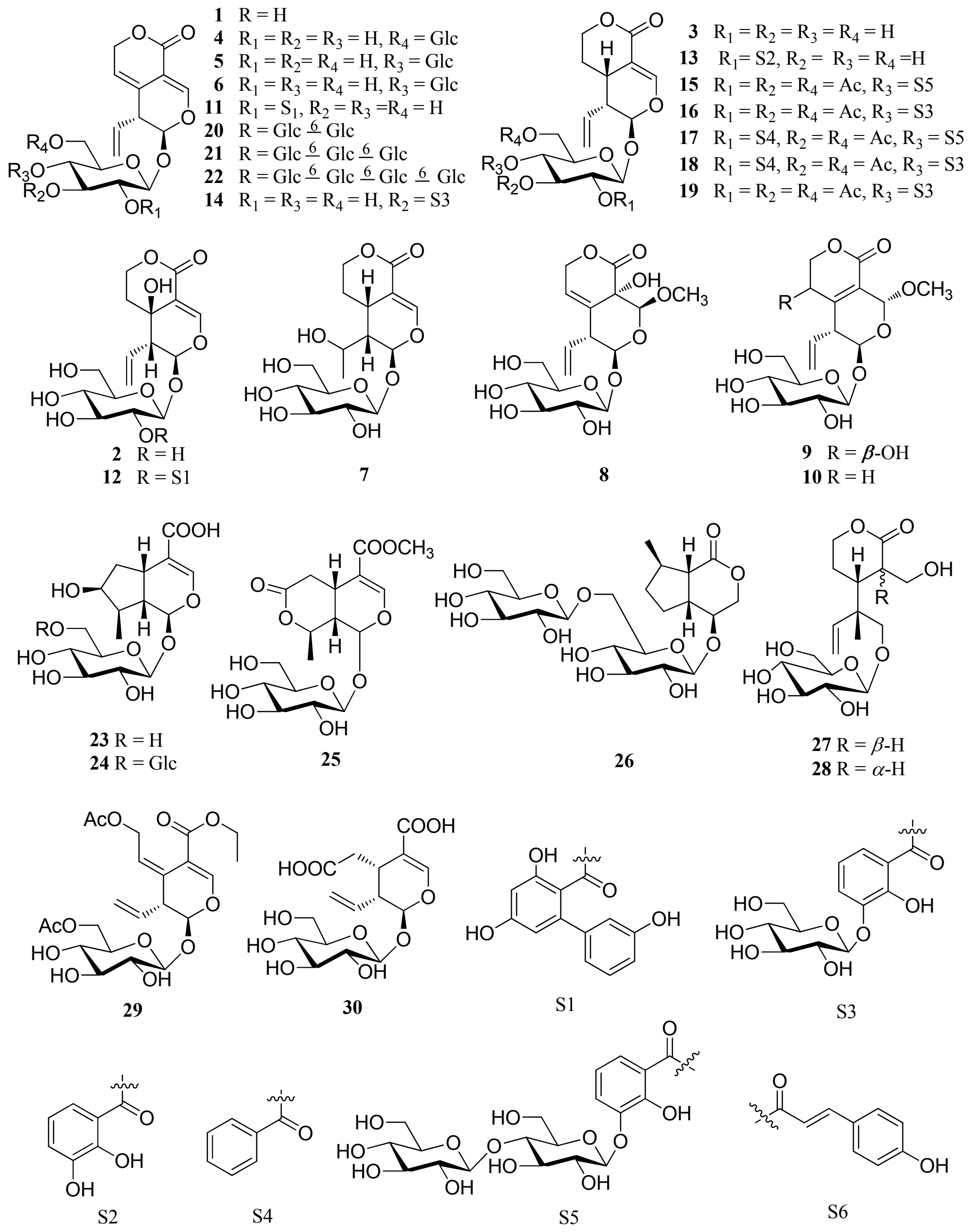

**Fig. 1.** Iridoids from "Long-Dan"

induced liver damage, but also offer protection against immune-mediated liver damage.[24–26] Studies showed that gentiopicroside could decrease the serum ALT and AST levels, and increase the liver GSH-Px activity in the mice treated with $CCl_4$. It could also promote the secretion of bile and increase the concentration of bilirubin in the bile.[27,28]

**4.2 Anti-inflammatory and Analgesic Effects:** The aqueous extract of Radix Gentianae had a remarkable inhibition of picryl chloride (PC) induced contact dermatitis and the inhibitory intensity of which was equal to that of pred-nisolone at the experimental doses. The extracts, however, did not influence both inflammatory reactions induced by xylene in ear and egg white in footpad.[29] Comparison analyzes on pharmacological function of the aerial and underground parts of Radix Gentianae showed that the anti-inflammation effect of aerial part was better than that of the underground one.[30] The essential oils of *G. scabra* exhibited a good performance of inhibiting NO production and xylene-induced ear edema in mice with a dose-dependent manner.[31] Ethanolic extract of "Guan-Long-Dan" showed anti-inflammatory and analgesic effects,[32] can protect the lung by inhibiting the expression of COX-2, reducing the production of PGE2 and promoting the generation of IL-10, in curing acute lung injury (ALI).[33] Gentiopicroside (**1**) was also reported to not only

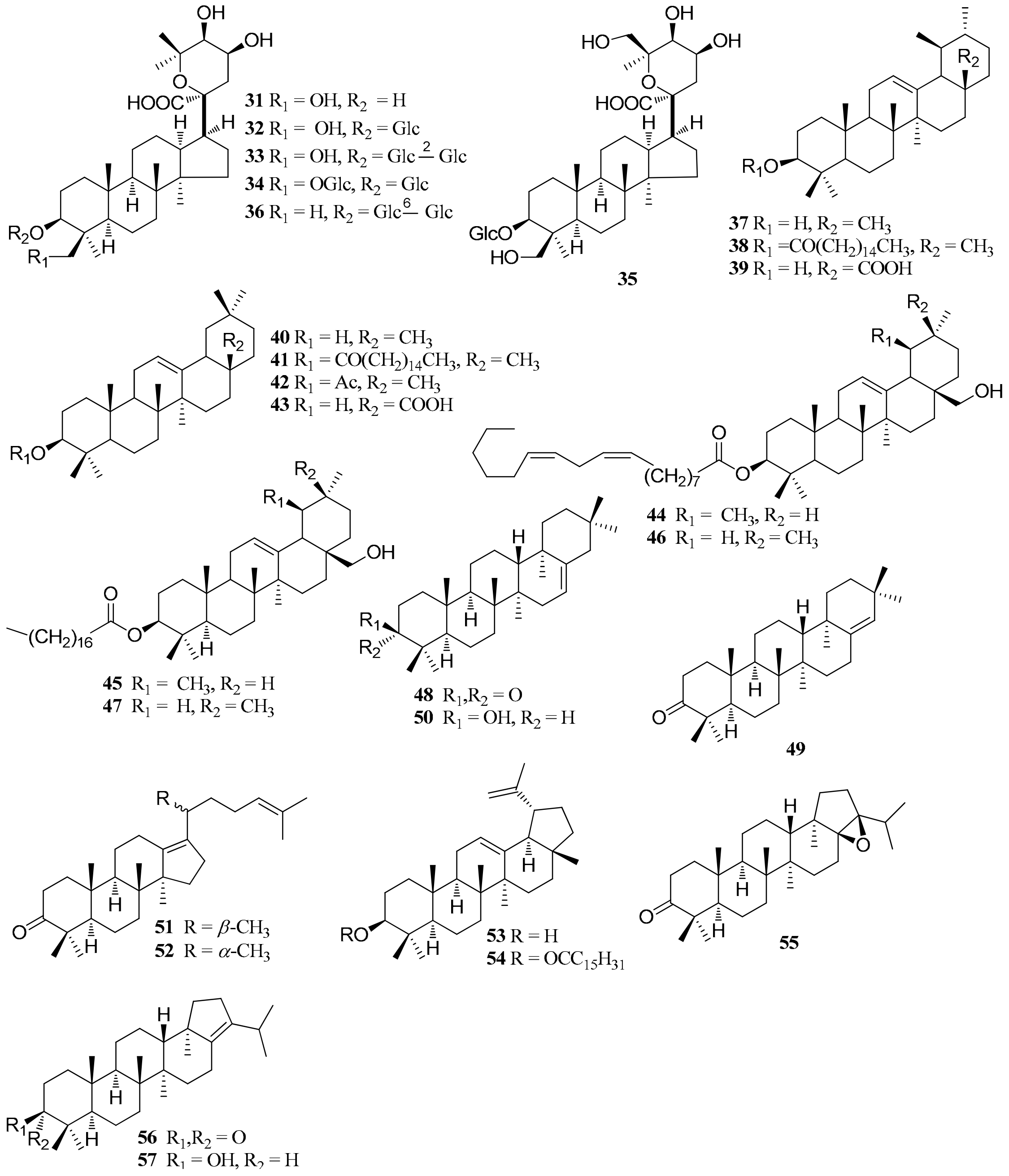

**Fig. 2.** Triterpenes from "Long-Dan"

inhibit the auricular edema and decrease the permeability of celiac blood capillary, but also reduce the paw swelling induced by carrageenan and zymosan A.[34]

**4.3 Antiproliferative Effecs:** The water extracts of *G. scabra* had inhibitory effects on sarcoma 180 in mice.[35] Two compounds, chirat-16-en-3-one (**48**) and chiratenol (**50**), from *G. scabra* showed moderate anti-tumor effect against Hela and HepG-2 lines.[13]

**4.4 Antimicrobial Effects:** 2,3-Dihydroxy benzoic acid methyl ester 3-*O*-*β*-D-glucopyranosyl-(1→6)-*β*-D-glucopyranoside (**84**) showed inhibitory activities against the growth of the plant pathogen *Peronophythora litchi* and *Glorosprium musarum*.[20] The triterpenoid glycosides, gentirigeosides A (**32**), C (**34**), and E (**36**) displayed antifungal activity against the plant pathogen *Glomerella cingulata*.[36] While experimental results showed that the aerial parts and the underground part of *Gentiana spp.* inhibited the growth of *Escherichia coli*, *Staphylococcus aureus*, and *Proteus* in different degrees.[30]

**4.5 Other Effects:** It was reported that 2-hydroxy-3-

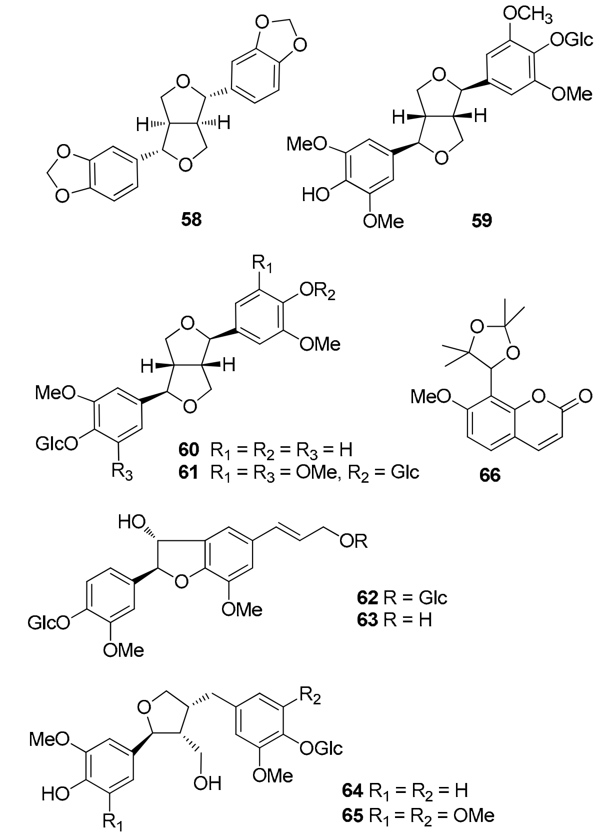

**Fig. 3.** Lignans and coumarin from "Long-Dan"

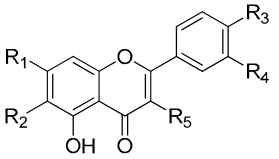

**67** $R_1$ = OGlc, $R_2$ = Glc, $R_3$ = OH, $R_4$ = $R_5$ = H
**68** $R_1$ = OH, $R_2$ = Glc, $R_3$ = OGlc, $R_4$ = $R_5$ = H
**69** $R_1$ = $R_3$ = $R_4$ = OH, $R_2$ = Glc, $R_5$ = H
**70** $R_1$ = $R_4$ = OH, $R_2$ = Glc, $R_3$ = OGlc, $R_5$ = H
**71** $R_1$ = $R_3$ = $R_5$ = OH, $R_2$ = $R_4$ = H

**Fig. 4.** Flavonoids from "Long-Dan"

methoxybenzoic acid glucosyl ester (**87**) could prevent mice from the PAF-induced death at a dose of 300 $\mu$g.[19] Gentisides A–K (**72–82**) from *G. rigescens* induced the growth of PC12 cells. Among them, gentiside C (**74**) showed a significant neuritogenic activity at 1 $\mu$M against PC12 cells comparable to that seen for the best nerve growth factor (NGF) at a concentration of 40 ng/mL. While gentiside C possessing the shortest alkyl chain length exhibiting the highest neuritogenic activity among all of the gentisides indicated that the length of the alkyl chain was important for the activity.[17,18]

*G. triflora* polysaccharide enhanced thymus index and spleen swelling index, increased carbon clearance index, and significantly higher HC50, thus enhancing the specific and non-specific immune function of mice.[37] What's more "Long-Dan" was also used in the treatment of hypothyroid conditions.[38]

## 5 Quality Control

The experimental results demonstrated that gentiopicroside was the main effective components of "Long-Dan", therefore it ought to be the most important index in evaluating the quality of these herbs. The Chinese Pharmacopoeia requires that the content of gentiopicroside should be not less than 2%.[1] Thin-layer-chromatography (TLC), reverse phase high-performance liquid chromatography (Rp-HPLC), high performance capillary electrophoresis (HPCE) methods etc., have been used for the determination of the content of gentiopicroside in these herbs.[39–41] It's reported that there were many factors influencing the quality control of "Long-Dan", such as species, growing place, growing periods, collecting season, processing and storing, etc.[26,42] According to the HPLC analysis of Jiang et al, the content of gentiopicroside in "Long-Dan" from different growing places ranged from 0.89% to 6.47% with an average of 2.97%. *G.* triflora possessed the highest content (6.47%) of gentiopicroside in all the samples and content in *G. manshurica*, *G. scabra*, and *G.* rigescens were 4.19%, 2.31%, and 6.28%, respectively. Wei et al. determined the content of gentiopicroside in 38 samples of Radix Gentianae from different habitat and the results indicated that the content of gentiopicroside varied considerably from place to place.[43] Thus, it should be combined all the factors to give a rational quality evaluation of "Long-Dan".

## 6 Concluding Remarks

Four different *Gentiana* species distributed in different areas of China have been recorded in the Chinese Pharmacopoeia to be used as the original plants of "Long-Dan". Though gentiopicroside together with swertiamarin, sweroside, and loganic acid, was the major component, different origins resulted in different minor constituents. So far, a large number of phytochemical studies have been carried on *G. scabra* and *G. rigescens*, however, it still remains to be exploited for the chemical constituents of *G. manshurica* and *G. triflora*. Previous reports on *G. scabra* and *G. rigescens* yielded the similar main chemical components. However, different minor compounds were also identified. Modern pharmacological researches on these four different species demonstrated the significant hepatoprotective effect of "Long-Dan" and other bioactivities, which are coincide with modern clinical application, while the bioactivities of minor constituents were pressing for research to get more different properties of these four different origins. Therefore, it needs more work on the chemical and pharmacological comparison of the original plants of "Long-Dan", especially those of *G. manshurica* and *G. triflora*. Whether the minor constituents exert any appreciable influence on the pharmacological activities, it should be further studies on, especially the discrepant chemical constituents. In brief, we should pay much attention and attach more importance to these traditional Chinese medicines, which have been used widely, but have more than one origin, and integrate various new technologies into traditional medicine research in the future studies, in order to understand them comprehensively and utilize them reasonably and scientifically.

## Acknowledgments

This work was supported by Science and Technology Planning Project of Yunnan Province (2010CD106), the 973

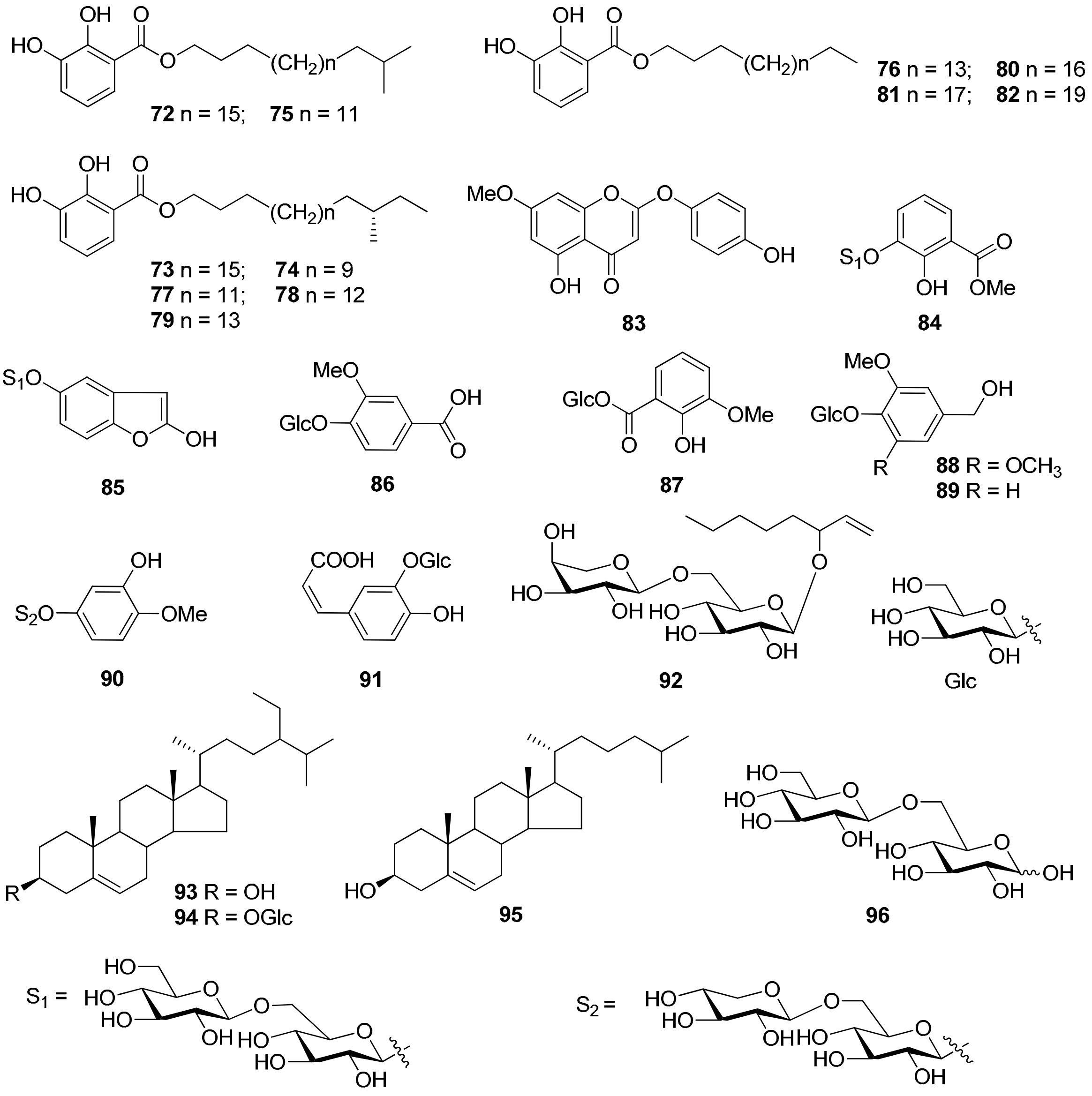

**Fig. 5.** Other compounds from "Long-Dan"

Program of Ministry of Science and Technology of China (2011CB915503). the State Key Laboratory of Phytochemistry and Plant Resources in West China, Chinese Academy of Sciences (P2010-ZZ03) and The Fourteenth Candidates of the Young Academic Leaders of Yunnan Province (Min XU, 2011CI044).

**Open Access** This article is distributed under the terms of the Creative Commons Attribution License which permits any use, distribution, and reproduction in any medium, provided the original author(s) and source are credited.
